# Measles: A Narrative Review of Current Therapeutic Options and Implications for the Future

**DOI:** 10.3390/v18080816

**Published:** 2026-07-25

**Authors:** Amalia Papanikolopoulou, Ilektra Tseliou, Helena C. Maltezou

**Affiliations:** 1 Naval and Veterans Hospital of Athens, 70 Democritus Street, 11521 Athens, Greece; amaliapapaniko@yahoo.com; 2 Department of Internal Medicine, General Hospital for Chest Diseases “Sotiria”, 152 Mesogeion Ave, 11527 Athens, Greece; ilektrat@yahoo.com; 3 Directorate for Research, Studies, and Documentation, National Public Health Organization, 3-5 Agrafon Street, 15123 Athens, Greece

**Keywords:** measles virus, antivirals, ribavirin, remdesivir, interferon, steroids, investigational agents

## Abstract

Despite the availability of a safe and effective measles vaccine for several decades, measles remains a significant cause of morbidity and mortality worldwide, causing 95,000 deaths in 2024 alone. Currently, there is no licensed therapeutic agent against measles, and treatment is only supportive. This is a review of articles published from 2015 to 3 June 2025, on therapeutic agents used or under investigation against measles. Ribavirin has been used in severe measles cases; nevertheless, there are no randomized clinical trials so far. New delivery systems, such as polymer-based formulations and nanoparticles, have markedly increased ribavirin’s efficacy, while cytotoxicity remained low. Viral membrane fusion (F) protein inhibitors and viral RNA polymerase inhibitors are promising next-generation therapeutics against measles and have been tested in animal models with good results. Lastly, interferons (IFNs) are key mediators of the innate immune response against measles, particularly for controlling brain infection. There are few reports indicating that repeated remdesivir courses, as well as intraventricular or intrathecal ribavirin or IFN, may temporarily stabilize the neurologic status of patients with subacute sclerosing panencephalitis. Intracranial administration of protein F inhibitors also demonstrated promising results in mice. Although several antiviral agents and natural products exhibit in vitro efficacy against measles virus, further research is required to determine their safety, pharmacological properties, and clinical efficacy before they can be considered therapeutic options. Overall, the development of antiviral agents against measles has been extremely slow. Therapeutic agents against measles are urgently needed.

## 1. Introduction

Measles virus (MeV), an enveloped, single-stranded, negative-sense ribonucleic acid (RNA) virus of the genus *Morbillivirus* of the *Paramyxoviridae* family, is a highly contagious virus [1,2]. The virus is transmitted from person to person, mainly through the airborne route, but also through direct contact with respiratory secretions [1,3]. Measles has a long incubation period (range: 7–23 days) [2]. After the virus enters into the respiratory tract, it spreads to lymphoid and non-lymphoid organs and causes an acute systematic infection [4]. Clinical signs range from the typical febrile exanthematous disease to life-threatening pneumonia and encephalitis, which disproportionately affect young children, older adults, and immunocompromised people [2,3,5,6,7,8].

Before the introduction of measles vaccine into routine vaccination programs in the 1960s, measles caused an estimated 2.6 million deaths per year worldwide [9]. According to estimates by the World Health Organization (WHO), measles vaccination averted approximately 59 million deaths from 2000 to 2024 [9]. Nevertheless, measles remains a significant cause of morbidity and mortality worldwide, and resurges even in countries with longstanding vaccination programs, mainly due to inequalities in access to vaccinations, vaccine hesitancy, and disruption of vaccinations during the coronavirus disease 2019 (COVID-19) pandemic [10]. According to the WHO, measles caused 95,000 deaths in 2024 alone, mainly among young children [9]. Currently, there is no licensed antiviral therapy against measles, and treatment is only supportive. Given the onset of decline in MeV loads by the time of eruption of skin rash, antivirals most probably will not address uncomplicated cases, but rather target immunocompromised individuals and patients with severe complications, including patients with subacute sclerosing panencephalitis (SSPE). This article reviews the recent literature on therapeutic agents for measles published from 2015 and onward, with a focus on new investigational agents.

## 2. Methods

This is a structured narrative review. The PubMed database was searched for articles published from 2015 to 3 June 2025, using combinations of the word “measles” with each of the following words separately: “antivirals”, “ribavirin”, “interferon”, “remdesivir”, “steroids”, “investigational agents”, and “plants”. Articles presenting data related to the treatment of measles were selected for inclusion, based on their relevance. Articles solely on vitamin A and plasma exchange were not considered. Duplicate articles were excluded. All (three) authors participated in the search and selection procedure, retrieval of data, synthesis of findings, and writing of the review.

The abstracts of 1016 articles identified through the first search round were screened, and 149 articles were selected for full reading. A total of 74 articles were included in this review [11,12,13,14,15,16,17,18,19,20,21,22,23,24,25,26,27,28,29,30,31,32,33,34,35,36,37,38,39,40,41,42,43,44,45,46,47,48,49,50,51,52,53,54,55,56,57,58,59,60,61,62,63,64,65,66,67,68,69,70,71,72,73,74,75,76,77,78,79,80,81,82,83,84].

## 3. Results

The findings of the reviewed articles are presented by therapeutic agent. Table 1 shows in vitro studies, Table 2 shows animal studies, Table 3 shows articles presenting data on the treatment of humans with acute measles, and Table 4 shows data from articles on the treatment of humans with SSPE.

### 3.1. Ribavirin

Ribavirin is a synthetic guanosine analog with broad-spectrum antiviral activity against specific deoxyribonucleic acid (DNA) and RNA viruses, including MeV [11]. The activity of ribavirin is accomplished via inhibition of inosine 5′-monophosphate dehydrogenase. Once inside cells, ribavirin is rapidly converted into phosphorylated forms suppressing viral RNA polymerases and reducing intracellular guanine reserves, disrupting viral replication through a mechanism known as lethal mutagenesis [11].

Ribavirin’s antiviral activity against MeV has been demonstrated in Vero cells, and its efficacy may be improved through complexation with delivery systems such as polymer-based formulations and gold or silver nanoparticles [12]. Hinton et al. achieved the increase of the antiviral activity of ribavirin through chemically linking the drug to large polyanionic polymers [13]. Particularly, the design combined both the antiviral effects of the polymer backbone and the intracellular delivery of ribavirin as a released drug payload. The produced multivalent structure increased interactions with viral particles or host-cell surfaces, improving inhibitory activity. In addition, the macromolecule’s antiviral activity most likely interferes with viral entry and infection process [13].

Markedly greater antiviral activity, compared to free ribavirin, has also been shown with ribavirin-gold nanoparticle conjugates, which act through enhancing interactions with biological targets, and therefore improve cellular uptake and delivery, while exhibiting low cytotoxicity [12,14]. For instance, at a concentration of 99.5 µg/mL, ribavirin-nano gold particles reduced MeV infectivity by 78.1%, whereas free ribavirin at a concentration of 500 µg/mL achieved only 25.4% viral reduction [14]. The strongest antiviral effect occurred in post-infection treatment experiments, implying that ribavirin-nano gold particles may primarily inhibit the intracellular stages of MeV replication rather than blocking viral attachment or entry [14].

Clinical data on ribavirin’s efficacy in measles are limited and somewhat contradictory, so its use is generally considered only in severe or complicated cases. It has been studied and used to treat patients at high risk for complications, including infants and children aged <5 years, adults aged >20 years, pregnant women, and immunocompromised people with hematological malignancies or human immunodeficiency virus infection [15]. In a study of three immunocompetent adults with measles complicated by pneumonia and respiratory failure, all of whom received high-dose vitamin A for three days, intravenous ribavirin was administered to two of them [16]. Clinical improvement and reversal of respiratory compromise were observed after treatment, supporting a potential therapeutic role for intravenous ribavirin and vitamin A in severe measles pneumonia [16]. In another case report, the administration of intravenous ribavirin with high-dose vitamin A to one patient with severe measles complicated by pneumonia, encephalitis, and multiorgan dysfunction, was associated with complete clinical recovery of respiratory, neurological, cardiac, renal, ophthalmic, and digestive functions [17]. The authors proposed that early ribavirin administration may reduce morbidity and mortality in severe measles cases, although they acknowledged the lack of standardized treatment guidelines and randomized clinical trials [17].

Because clear guidelines for the treatment of severe measles across different patient populations are lacking, ribavirin dosing strategies, treatment outcomes, and reported adverse effects vary substantially among studies. In the previous studies, the combined treatment administered included intravenous ribavirin for 5 days, at 2 g as a loading dose and 1 g four times a day as maintenance dose, plus intramuscular vitamin A at 200,000 IU per day for three [16] or four days [17]. The combined treatment was generally well tolerated. Early ribavirin treatment has also been described in an adult with severe measles pneumonitis [18]. Administration of oral ribavirin was well tolerated and was followed by reversal of respiratory compromise, suggesting that early ribavirin treatment may have a potential role in severe measles-associated respiratory disease, although robust evidence remains limited. Treatment schedule consisted of oral ribavirin at 400 mg twice daily for four days with good tolerance [18].

In patients with SSPE, a rare, progressive, fatal degenerative brain disorder caused by the persistence of a mutated wild-type MeV which leads to SSPE in the central nervous system (CNS), treatment remains largely experimental [19,20]. Oral administration of ribavirin has no effect on SSPE patients, probably because cerebrospinal fluid (CSF) concentrations remain low [21]. Nevertheless, there are several clinical reports that indicate that ribavirin has the potential to reduce antibody titers in CSF without causing side effects, particularly when used in conjunction with interferon-α (IFN-α) [6]. Miyazaki et al. developed a continuous intraventricular ribavirin delivery strategy using a subcutaneous infusion pump for patients with SSPE to achieve stable concentrations within the target therapeutic range of 50–200 μg/mL, which is considered sufficient to inhibit virus replication and potentially slow disease progression, with treated patients surviving beyond five years despite advanced disease [22].

Despite these recent findings, well-structured double-blind randomized controlled trials are lacking, and ribavirin is not approved for the treatment of measles. Its administration in severe cases is off-label, both in the United States and within the European Union. Oral ribavirin is commercially available, but intravenous ribavirin requires an emergency use of investigation drug application.

### 3.2. Interferons

IFNs represent key mediators of the immune response against MeV infection, limiting viral replication and promoting the activation of adaptive immunity. However, MeV has evolved several mechanisms to suppress the IFN response, including nonstructural proteins V and C, which inhibit the recognition of viral RNA and subsequently block the production of type I IFNs [24]. In particular:

C protein: suppresses the production of defective interfering particles, which normally induce strong type I IFN production [25].

V protein: inhibits type I interferon responses and may also interfere with type III IFN responses [26,27,28].

These factors account for the delayed activation of the immune system as well as the lack of a robust innate immune response. It has been shown that genetic ablation of the C and V proteins resulted in enhanced production of type I and III interferons, leading to a significantly more effective clearance of the virus in lung epithelial cells [28].

In the CNS, the protective role of INFs remains highly significant. Type I IFN response is crucial not only in the control of CNS infection but also in the early permissiveness of glial cells to MeV [23]. Additional findings from genetically modified mice models complement the above research, revealing that IFN-γ protects neural stem and progenitor cells from the harmful effects of MeV infection in the neonatal brain without promoting their proliferation [29].

These findings highlight the critical role of IFN signaling in controlling MeV infection and provide a rationale for investigating exogenous IFNs as a potential therapeutic strategy. Indeed, IFN pathways have been shown to be dysregulated in SSPE patients [30,31]. In this context, the administration of exogenous IFNs may serve as a viable therapeutic strategy. Intraventricular and intrathecal IFN-α therapy has been administered to adolescent patients with SSPE, followed by neurologic stabilization [32,33,34]. Studies are needed to further investigate the potential role of IFN in patients with SSPE. Nevertheless, although several antiviral and immunomodulatory agents are available, the optimum treatment has not been established yet, partly due to the absence of common diagnostic and clinical standards for SSPE [35].

### 3.3. Remdesivir

Remdesivir is a nucleoside analog prodrug, exhibiting extensive in vitro antiviral efficacy against various RNA viruses, including MeV. Specifically, against MeV, remdesivir inhibited viral replication at sub-micromolar concentrations, indicating strong in vitro activity [36,37]. Recently, remdesivir has been evaluated in non-human primate models, such as rhesus macaques, for its effects on MeV. The findings of these animal studies suggest that post-exposure prophylaxis (PEP) with remdesivir started early, typically within three days after exposure or infection, can reduce MeV RNA levels during the 12-day treatment period and also limit viral replication in peripheral blood mononuclear cells. Despite this suppression, viral RNA rebounds after discontinuation of treatment, and infectious virus can still be recovered, indicating that remdesivir does not fully clear the virus [37,39].

Measles normally causes loss of preexisting antibodies to other pathogens by damaging immune memory cells, a phenomenon called “immune amnesia”. Macaques treated early post-exposure with remdesivir retained more antibodies against previously encountered viruses than untreated animals [39]. However, remdesivir PEP reduced the magnitude and breadth of the antibody response to MeV itself, with lower measles-specific antibody levels and fewer measles epitopes recognized, potentially affecting long-term immunity to measles [39]. Clinically, remdesivir PEP did not improve disease symptoms or outcomes with no meaningful reduction in rash, fever, lymphadenopathy, or overall disease severity and did not prevent measles-associated lymphopenia, a hallmark of measles immune suppression. These findings suggest that remdesivir PEP could be useful to mitigate measles-induced immunosuppression and limit secondary infections [38]. Furthermore, late treatment with remdesivir, initiated around the onset of measles rash (about 11 days post-infection), has limited impact on viral load and immune memory preservation [38]. Unlike PEP, viral RNA clearance from lymphoid tissue and clinical outcomes are not improved by late treatment, and infectious virus can be detected, indicating incomplete viral suppression [39]. These findings suggest that remdesivir should be administered early to limit immune amnesia and viral replication effectively, while late treatment offers minimal benefit. Pharmacokinetic studies conducted in rhesus monkeys have shown that remdesivir and its derivative active nucleosides can reach the brain and potentially could be used in the treatment of SSPE [21,40]. There are very few case reports regarding remdesivir treatment and SSPE [41,42]. Evidence from these studies suggested that remdesivir may temporarily reduce viral activity, stabilize neurological decline, or improve some clinical symptoms in SSPE patients. However, these findings were generally incomplete and not permanent [41,42]. In one case, the administration of multiple courses of remdesivir was associated with transient clinical improvement without inducing viral escape mutants but ended with a fatal outcome [41].

### 3.4. Steroids

Corticosteroids, such as dexamethasone, are powerful modulators of inflammation and may reduce secondary neuronal injury by attenuating inflammation-associated complications, including cerebral edema and immune-mediated tissue damage [43]. Nevertheless, current clinical evidence regarding their efficacy in viral encephalitis remains inconclusive [43]. The potential utility of corticosteroids is underscored in the case of a 30-year-old male with severe measles infection complicated by pneumonia, encephalitis, and acute disseminated encephalomyelitis [44]. Following a combination of intravenous immunoglobulin, high-dose corticosteroid therapy with methylprednisolone pulse regimen, vitamin A, and plasma exchange, the patient had a favorable clinical outcome [44]. However, data from a retrospective study of 113 patients hospitalized with measles did not find any clinical or biochemical benefit of corticosteroid use [45]. Particularly, no significant differences were observed regarding complication rates and length of hospital stay between patients who received corticosteroids and those who did not [45]. Notably, the corticosteroid-treated group had lower lymphocyte and platelet counts and higher C-reactive protein levels, but higher self-perceived recovery rate compared to untreated patients [45]. Furthermore, in a meta-analysis of 15 studies with 1836 patients with viral encephalitis, although two studies suggested potential clinical benefits such as faster resolution of symptoms or reduced mortality, most studies did not find a clear therapeutic advantage [43]. Overall, this meta-analysis found an increased risk of selection and allocation bias, primarily due to the non-randomized assignment of more severely ill patients for corticosteroid treatment [43]. In addition, limited data regarding adverse effects, together with the heterogeneity and small sizes of studied populations, significantly limit the generalizability of the findings of this meta-analysis [43]. While current evidence does not support the routine use of corticosteroids in measles-associated encephalitis [43], emerging data from case reports [46] suggest a potentially beneficial role for corticosteroids in measles-associated pneumonitis.

### 3.5. Investigational Agents

#### 3.5.1. Agents Targeting the Viral Fusion

Viral membrane fusion (F) protein is one of the most promising antiviral targets as its activation and conformational change enables the entry of viral RNA to the cytoplasm of the host cell [47]. Halting membrane fusion not only inhibits virus entry but also cell-to-cell spread through multinucleated cell formations called syncytia, a hallmark of many paramyxoviral infections [47]. Peptide-based fusion inhibitors that prevent virus entry and syncytia formation are among the most advanced therapeutic approaches against measles [48,49,50]. Bovier et al. designed a dual-action antiviral peptide to attack two different functional regions of the F protein in a cotton rats’ model. The two inhibitors act synergistically against the activation of MeV membrane F protein, while protecting the rats from infection [51].

Fusion inhibitors may be especially important for CNS complications. Increased fusion capacity, as shown by hyperfusogenic measles variants, enhances the ability to infect and promote viral spread in CNS, including SSPE [6]. Interestingly, there is heterogeneity in susceptibility to MeV among different CNS cell populations [52]. Some synthetic novel peptides derived from the F protein of the MeV demonstrated high in vitro antiviral activity against both MeV and mutated MeV strains which lead to SSPE [53]. For instance, intracranial administration of one of the synthetic peptides increased the survival rate, from 0% to 67%, in mice infected with mutated MeV strains which lead to SSPE [53]. Fusion inhibitors are currently the only antiviral class that has been tested against both the wild-type and hyperfusogenic variants bearing specific virus mutations identified in CNS infections [51]. Lastly, Reynard et al. developed and standardized a respiratory delivery platform to deliver fusion inhibitor peptides to the upper and lower respiratory tract of macaques, a model that closely mimics natural measles transmission [54].

#### 3.5.2. Agents Targeting RNA Polymerase

The viral RNA polymerase complex is another attractive target for antiviral design against the MeV. Measles polymerase regulation depends not only on catalytic activity but also on structural remodeling by proteins [55]. Recent discoveries through structural biology and cryo-electronic microscopy have clarified its architecture, identified critical protein–protein interactions, and revealed conformational states essential for replication [56,57,58,59]. For instance, the role of cofilin, regarding replication and formation of the MeV ribonucleoprotein complex, has been investigated [59]. MeV infection increases the amount of the inactive form of cofilin, which promotes stabilization of actin filaments and contributes to the intracellular environment needed for replication [59]. It is suggested that host proteins involved in actin regulation could be potential antiviral targets [59]. These recent discoveries have enabled nucleoside analog development, non-nucleoside allosteric inhibitors, and polymerase-complex disruptors as promising next-generation therapeutics [60,61,62,63,64].

Non-nucleoside antiviral compounds that inhibit viral RNA polymerases have also been investigated. Non-nucleoside inhibitors bind at a regulatory region of the polymerase complex, which is different than the active catalytic site of nucleosides. This indirectly alters polymerase function and suppresses RNA synthesis but also prevents progression through the replication cycle [65]. Among the most advanced candidates identified to date are the anti-measles small molecules AS-136a and ERDRP-00519, both of which function as polymerase inhibitors. These compounds have the potential to achieve strong antiviral efficacy in vivo [65]. Particularly, the ERDRP-00519 blocks all phosphodiester bond formation, which locks the viral polymerase in a pre-initiation conformation, thereby preventing viral RNA synthesis [66]. Treatment of animals with ERDRP-0519 prevented the onset of symptoms while allowing the development of protective immunity [67,68]. In squirrel monkeys, treatment initiation at the onset of clinical signs reduced MeV shedding, which may support outbreak control [68].

Another approach to inhibit the function of the RNA-dependent RNA polymerase complex is through monoclonal antibodies, such as 1D11-IgA against phosphoprotein of MeV [69]. Although the effect of 1D11-IgA on viral replication was modest, this study provides further experimental evidence that phosphoprotein is a potential target that could be inhibited through different epitopes via different drug molecules [69].

#### 3.5.3. Favipiravir

Favipiravir is a small molecule that has demonstrated antiviral activity against MeV and the mutated MeV strains which lead to SSPE [70]. The potential mechanism of favipiravir for suppressing viral replication includes its binding to the active sites of MeV RNA polymerases [71]. Computer-based simulations indicate that it can disrupt RNA replication or induce replication errors, which require further laboratory and clinical validation [72].

#### 3.5.4. Other Investigational Agents

The antiparasitic drug nitazoxanide was evaluated against MeV and reduced its replication in cell culture assays [73]. Antiviral effects increased with higher concentrations of the drug, demonstrating dose-dependent activity. Its major action occurred at the post-entry stage of infection, interfering with later stages of viral replication, but also disrupting the function of the viral F protein and reducing syncytia formation [73].

The antiviral activity of the amphibian antimicrobial peptide Hylin-a1 against several respiratory viruses, including MeV, has been investigated [74]. The results suggested interference with viral attachment, membrane fusion, and entry into host cells. In MeV experiments, treatment with Hylin-a1 reduced viral infection and replication in cultured cells and the antiviral effect was dose-dependent [74].

Numerous host factors, which are necessary for viral replication, have also emerged as therapeutic targets and host-directed therapies. In a lymphocytes MeV infection model, it has been shown that the sphingosine kinase II inhibitor and the acid ceramidase inhibitor ceranib-2 effectively suppressed viral replication, reducing the titers of newly synthesized viruses by approximately 1 log (90%) in peripheral blood cells [75]. Further research showed that both inhibitors suppress protein synthesis pathways used by the virus [76].

De novo pyrimidine biosynthesis is a key metabolic pathway exhibiting broad-spectrum antiviral activity and immune-stimulatory properties. A new class of pyrimidine biosynthesis inhibitors has been designed, which inhibits de novo pyrimidine biosynthesis, amplifies IFN expression in cells, and modulates the innate immune response in MeV-infected cells [77]. Another important host pathway is the phosphorylation of serine 51 on the α subunit of eukaryotic translation initiation factor 2 (eIF2α). The guanabenz derivative Sephin1 has shown a dose-dependent inhibition of MeV replication in cells [78]. Lastly, the inhibitor ESI-09 showed a broad-spectrum antiviral activity against both RNA and DNA viruses, including MeV [79]. This was achieved through interference with viral internalization and cell-to-cell transmission, but also inhibition of entry pathways and reduction in syncytia formation [79]. Although viral titers decreased substantially (often by 3–4 log reductions), there were no significant effects on cell viability at tested concentrations [79].

### 3.6. Plant Medications

Natural products from terrestrial plants, aquatic plants, algae, and nanotechnology-based formulations have demonstrated promising in vitro antiviral activity against the MeV, primarily by interfering with viral entry, directly inactivating viral particles, or suppressing viral replication [80,81,82,83,84].

The plant-derived naphthoquinone droserone, a natural product found in dicotyledonous plants, showed potent inhibition of MeV infection at low micromolar concentrations (50% of virus reduction at approximately 2 μM of droserone) by targeting an early stage of viral entry, although its mechanism and therapeutic applicability remain unclear [80]. Other tested naphthoquinone derivatives, but also hydroxylated synthetic analogs, were either more cytotoxic or not as effective as droserone [80].

Similarly, polyphenol-rich extracts from the seaweeds *Ecklonia arborea* and *Solieria filiformis* exhibited remarkable virucidal activity and high selectivity indices, suggesting that marine organisms may represent valuable sources of anti-measles agents [81]. The tested polyphenol-rich extracts act mainly by inactivating the viral particle. Interestingly, both extracts demonstrated significant efficacy and minimal cytotoxicity when compared to ribavirin [81].

Extracts from *Nuphar lutea* demonstrated a different mode of action by reducing the expression of essential viral proteins and completely inhibiting the phosphoprotein P, which is crucial for viral replication. Reducing the expression of the latter protein is expected to effectively inhibit MeV replication and therefore represent a new therapeutic target [82].

Nanotechnology-based approaches have also shown therapeutic potential. Gold nanoparticles synthesized using *Allium sativum* (garlic) extract displayed stronger antiviral and virucidal effects against MeV than their precursor components, either garlic extract or chloroauric acid alone [83]. Gold nanoparticles could serve as a promising approach for the treatment of measles infections [83].

Lastly, a systematic review assessed the available evidence on African medicinal plants traditionally used for antiviral therapies and explored their potential role in the management of viral infections [84]. The review included 36 studies and evaluated 328 medicinal plant species, of which 127 exhibited significant antiviral activity against different viral pathogens. Notably, five plant species demonstrated activity against MeV. These findings suggest that African medicinal plants represent a valuable reservoir of bioactive compounds with potential for antiviral drug discovery. Nevertheless, the evidence remains largely preliminary and is based mainly on experimental studies, highlighting the need for further research before any clinical applications can be recommended [84].

## 4. Limitations

Our review has the following limitations: the use of a single database (PubMed), the absence of PRISMA guidelines, the lack of a risk of bias assessment, and the absence of evidence grading (GRADE/Oxford). Lastly, the search was terminated on 3 June 2025, which means that we may have missed some relevant articles published since then.

## 5. Conclusions

Although measles remains a significant cause of morbidity and mortality worldwide, the development of therapeutic agents has been extremely slow and, currently, no licensed treatment exists. The long incubation time of measles further compromises endeavors to develop therapeutic agents. Ribavirin has been used in severe clinical cases along with vitamin A; however, large randomized clinical trials are needed. Viral membrane F inhibitors and viral RNA polymerase inhibitors have also been tested in animal models with good results and emerge as promising next-generation therapeutics against measles. Overall, several antiviral agents and natural products possess anti-measles activity in laboratory models and may provide valuable compounds for future antiviral drug development. However, evidence remains limited to predominantly in vitro studies. Therefore, while these findings identify promising candidates and potential mechanisms of action, substantial research is required to determine their safety, pharmacological properties, and clinical relevance before they can be considered therapeutic options for measles.

## Figures and Tables

**Table 1:** In vitro studies of antiviral agents against MeV, 2015–3 June 2025.

Study [Ref]	Country	Characteristics	Therapeutic Agent	Findings	Comments
Hinton et al. [13]	Denmark	Vero cells	Polyanionic macromolecular ribavirin prodrugs	Highly effective, reducing the viral titer by >10-fold (TCID_50_ assay).	None
Ahmed et al. [14]	Egypt	Vero cells	Ribavirin-nano gold particles vs. ribavirin alone	Ribavirin-nano gold particles had higher activity with lower dose c/w ribavirin alone (78.1% viral reduction at 99.5 μg/mL).	Safety of ribavirin-nano gold particles.
Pfaller et al. [25]	USA	Analysis of de novo generation and sequence characteristics of copy-back defective interfering RNAs	C-protein knockout MeV	C-protein-deficient MeV generated higher levels of defective interfering RNAs, which reduced infectivity and suppressed virus replication.	The C protein limits defective interfering with RNA formation and preserves MeV replication efficiency.
Taniguchi et al. [28]	Japan	Lung epithelial cell line infected with MeV and mutant viruses	Genetic ablation of type I or type III IFN receptors and deletion of MeV V protein	Type I and III IFNs cooperatively restricted MeV growth, V protein suppressed IFN induction.	Control of MeV replication requires coordinated type I and type III IFN responses; V protein is a key IFN antagonist.
Lo et al. [36]	USA	Vero cells	Remdesivir, 1′-cyano substituted adenine nucleoside analog	Against MeV: remdesivir EC50/EC90 (μM)/[SI]: 0.037/0.073 Nucleoside analog EC50/EC90 (μM)/[SI]: 1.0/2.6.	Remdesivir and its nucleoside analog exhibit extensive in vitro antiviral efficacy against RNA viruses from various families, including MeV.
Schmitz et al. [37]	The Netherlands	Various cell lines	160 compounds were screened	21 compounds appeared active, the strongest were highly cytotoxic.	No clearly usable new antivirals identified.
Ader-Ebert et al. [48]	USA	Molecular mapping of the mAb-1347 epitope	mAb-1347	Inhibits virus cell entry and spread.	In mAb-1347, a receptor induces “head-stalk” conformational change prior to the “opening” of the central stalk section required for fusion activation. Mechanism other than blocking receptor binding.
Hirata et al. [50]	Japan	Vero cells	MEK35 (modified peptide inhibitor that blocks variants resistant to M1/M2)	Strong activity against M1 resistant MeV variant with EC50: 0.028 μΜ.	In this study a three-step fusion inhibition model is proposed, beyond the old idea that inhibitory peptides simply bind as static helices. Resistance disrupts the normal transition process involved in inhibitor binding, but MEK35 overcomes this barrier and maintains effective antiviral activity.
Bovier et al. [51]	USA	HEK-293T, Vero, and Vero-Slam/CD150 cells	Hybrid molecules combined a fusion inhibitor peptide and a lipopeptide	Strong inhibition of measles infection, mainly in membranes containing the F protein.	Dual-action antiviral peptide to target multiple functional regions of F protein resulted in a conjugated molecule with significantly enhanced ability to inhibit MeV fusion and infection.
Ferren et al. [52]	France	Vero cells	Wild-type vs. hyperfusogenic mutations	Hyperfusogenic viruses led to infection of significantly more cells; targeted cells seemed to be mainly neurons.	This is the first study to show that hyperfusogenicity helps the virus overcome antiviral defenses.
Watanabe et al. [53]	Japan	Vero cells and model of mice infected with mutated MeV, which led to SSPE	Synthetic peptides from the F protein of heptad repeat region of the MeV	The novel peptide M2EK inhibited the replication of mutated MeV, which led to SSPE by interfering with its fusion process in vitro and in vivo.	Potential candidate for SSPE treatment.
Cox et al. [56]	USA	Paramyxovirus RdRp complex structural forms	Distinct enzymatic activities of the RdRp complex and the highly dynamic protein–protein and protein–RNA interactions	Efficacy in animal models.	Promising approach to combine a substrate analog for RdRp with an allosteric inhibitor to maximize drug combination synergies and reduce the frequency of viral escape.
Patil et al. [61]	India	Vero cells	Thiopene derivative	Broad-spectrum antiviral activity.	None
Liu et al. [62]	USA	Vero cells	6′-fluoro-3-deazaneplanocin	Activity against MeV (EC50 < 0.36 μM; CC50 115 μM; SI > 320).	Can serve as prototype compound for antiviral agent design.
Liu et al. [63]	USA	Vero cells	Enantiomeric 3-deaza-1′,6′-isoneplanocins	Compounds 7a and 8a had anti-measles activity.	Enantiomeric 3-deaza-1′,6′-isoneplanocins as prototype for design of antiviral agents.
Haverkamp et al. [64]	USA	Vero cells	6′-fluoro-3-deazaneplanocin and its 3-bromo derivative	Efficacy against MeV (most effective: EC50 < 0.36 μM; CC50 115 μM; SI > 320).	None
Cox et al. [66]	USA	Purified MeV RdRp complexes and synthetic templates	ERDRP-0519, a pan-morbillivirus oral small molecule inhibitor	ERDRP-0519 inhibits all phosphodiester bond formation in de novo initiation of RNA synthesis at the promoter and RNA elongation by a committed polymerase complex.	Silences all viral RNA synthesis, locking the polymerase in pre-initiation conformation.
Hashimoto et al. [70]	Japan	MeV and mutated MeV strains which lead to SSPE on Vero-Slam cells	Favipiravir	EC50: 108.7 μM against MeV and 38.6 μΜ against mutated MeV strains which lead to SSPE	EC50 of famipiravir were similar to those of ribavirin.
Tokunaga et al. [71]	Japan	Vero-Slam cells	Favipiravir	Inhibited replication of all paramyxoviruses tested, with EC90 of 8 to 40 M.	None
Stelitano et al. [73]	Italy	Human kidney epithelial and Vero-Slam cells	NTZ (antiparasitic drug)	Antiviral activity at >20 μg/mL. NTZ potently impaired MeV replication, with IC50s of 3.23 μg/mL	Promising antiviral activity.
Chianese et al. [74]	Italy	Vero cells	Amphibian peptide Hylin-a1	Peptide Hylin-a1 interferes with viral attachment, membrane fusion process, destabilization of envelope integrity and reduction in infectivity.	None
Grafen et al. [75]	Germany	Human PBLs and human JAB cell line	Sphingosine kinase inhibitor II and acid ceramidase inhibitor ceranib-2	Efficient inhibition of MeV replication with titers reduced by ~1 log (90%) in PBLs and 70–80% in BJAB cells.	Sphingolipid metabolism as an approach for new therapeutic or immunomodulatory approaches against viral infections.
Lucas-Hourani et al. [77]	France	HEK-293T cells infected with a recombinant strain of MeV expressing luciferase	DD363	The IC50 (mean ± SD) was 11 ± 3 μM.	Multiple antiviral activities: 1. inhibition of de novo pyrimidine biosynthesis; 2. amplification of IFN expression in cells; 3. modulation of the innate immune response in infected cells.
Fusade-Boyer et al. [78]	France	HEK-293T cells infected with a genetically engineered MeV expressing the Firefly luciferase	Guanabenz derivative Sephin 1	Dose-dependent inhibition of MeV replication by Sephin1 (10-fold inhibition when Sephin1 was used at 40 μM, but cellular viability remained >75%).	Despite the necessity of administering Sephin 1 at a high dosage to achieve a high antiviral effect, the suppression of virus replication could not be achieved due to changes in cellular viability.
Boulton et al. [79]	Canada	Vero cells	ESI-09 inhibitor	Viral titers decreased substantially (often by 3–4 log reductions), no significant effects on cell viability.	Host-directed antiviral therapy as an alternative to conventional virus-specific drugs.
Lieberherr et al. [80]	Germany	Vero cells	Natural products of dicotyledonous plants: naphthoquinone droserone	The naphthoquinone droserone is a potent inhibitor of MeV when added during virus entry. Other derivatives are more cytotoxic, less effective.	Research tool to study MeV entry into cells.
Morán-Santibañez et al. [81]	Mexico	Vero cells	Polyphenol-rich extracts from seaweeds	Seaweeds, *Ecklonia arborea* and *Solieria filiformis* (Rhodophyta), had the highest SI (>3750 and >576.9, respectively) and significant efficacy with minimal cytotoxicity c/w ribavirin (SI: 11.57).	Polyphenol-rich extracts of seaweeds have virucidal effects.
Winer et al. [82]	Israel	Vero cells	Nuphar lutea extracts	Decrease in the MeV of P, N, and V proteins, reduction of released infective virus c/w untreated cells.	Activity against MeV, may represent new target for antiviral strategies
Meléndez-Villanueva et al. [83]	Mexico	Vero cells	Garlic extract-gold nanoparticles	Effective suppression of MeV replication with EC50 of 8.829 µg/mL and SI of 16.05.	Natural extracts combined with nanoparticles could be a promising approach for the treatment of measles.

MeV: measles virus; Ref: reference; TCID: tissue culture infectious dose; vs: versus; c/w: compared with; μg: microgram; mL: milliliter; USA: United States of America; RNA: ribonucleic acid; IFN: interferon; EC50: 50% effective concentration; EC90: 90% effective concentration; μΜ: micromolar; SI: selectivity index; mAb: monoclonal antibody; HEK: human embryonic kidney; F protein: fusion protein; SSPE: subacute sclerosing panencephalitis; RdRp: RNA-dependent RNA polymerase; CC50: 50% cytotoxicity concentration; NTZ: nitazoxanide; IC50: half-maximal inhibitory concentration; PBLs: peripheral B lymphocyte cells; log: logarithm; SD: standard deviation.

**Table 2:** Animal studies of antiviral agents against MeV, 2015–3 June 2025.

Study [Ref]	Country	Type of Study	No and Characteristics of Subjects	Therapeutic Agent	Findings	Comments
Welsch et al. [23]	France	In vivo experimental study	MeV-infected transgenic mice	Type I IFN signaling/IFNAR pathway	IFNAR signaling restricted virus spread in CNS.	Protective role of IFN-I signaling against measles CNS infection and encephalitis progression.
Van Nguyen et al. [24]	Japan	In vivo experimental study	Macaques infected with wild-type MeV (n = 3) or recombinant MeV (n = 3) expressing H protein of the Edmonston vaccine strain	Type I IFN	Infection with the recombinant MeV induced a strong IFN-α response.	The vaccine H protein contributes to MeV attenuation by promoting IFN responses.
Fantetti et al. [29]	USA	In vivo experimental study	Neonatal transgenic mice with neuron-restricted MeV infection	IFN-γ	Loss of IFN-γ reduced NSPCs, whereas NSPCs were preserved in IFNγ-competent mice.	IFN-γ protects neural stem cells from immune-mediated damage in the infected neonatal brain.
Peart Akindele et al. [38]	USA	Case-control study	15 macaques intratracheally infected with MeV: 9 were treated with remdesivir, 6 not treated	iv remdesivir for 12 days, either as: 1. PEP on day 3 (n = 6) 2. late treatment at day 11 (n = 3), vs untreated animals receiving similar diluent in each group (3 in each group)	Early PEP with remdesivir reduced MeV RNA levels during the 12-day period.	Viral RNA rebounds after stopping treatment. PEP did not improve disease symptoms or outcomes. Late treatment had limited impact on viral load.
Chan et al. [39]	USA	Case–control study	14 macaques intratracheally infected with MeV: 8 were treated with remdesivir, 6 not treated	iv remdesivir for 12 days, either as: 1. PEP beginning on day 3 (n = 5), 2. late treatment at day 11 (n = 3) vs not treated macaques (n = 6)	PEP limits antibodies’ loss caused by measles-induced immune amnesia. Clearance of viral RNA from lymphoid tissue and clinical outcomes did not improve by late treatment.	They measured longitudinal antibody reactivity to MeV and other viral peptides using plasma from MeV-infected macaques.
Whittwer et al. [68]	Germany	In vivo experimental study	Squirrel monkeys (6 treated c/w 6 untreated)	ERDRP-0519 at 50 mg/kg bid for two weeks (polymerase inhibitor)	Reduction in clinical signs of MeV infection in treated monkeys.	Reduced MeV shedding when treatment was initiated at the onset of symptoms.

MeV: measles virus; Ref: reference; IFN: interferon; IFNAR: interferon-alpha/beta receptor; CNS: central nervous system; H: hemagglutinin; USA: United States of America; NSPCs: neural stem and progenitor cells; iv: intravenous; PEP: post-exposure prophylaxis; vs: versus; RNA: ribonucleic acid; c/w compared with; mg: milligram; kg: kilogram.

**Table 3:** Articles presenting data on the treatment of humans with acute measles, 2015–3 June 2025.

Study [Ref]	Country	Type of Study	No and Characteristics of Subjects	Therapeutic Agent	Findings	Comments
Ortac Ersoy et al. [16]	Turkey	Case series	3 patients with severe measles pneumonitis (1 pregnant)	Iv ribavirin 2 g (LD) followed by 1 g × 4 (MD) (2 patients) and vitamin A 200,000 IU × 1 for 3 days (3 patients)	All patients recovered and discharged from the hospital.	Case 1: hepatic enzymes and serum bilirubin level increased on 4th day of ribavirin, drug discontinuation.
Bichon et al. [17]	France	Case report	1 patient with measles, complicated with pneumonia, acute encephalitis, pancreatitis, and corneous ulcer, associated with vitamin A deficiency	Ribavirin 2 g (LD) followed by 1 g × 4 (MD) for 5 days, vitamin A 200,000 IU × 1 for 4 days, ophthalmic vitamin A therapy	Patient recovered and was discharged from hospital.	Complicated pneumonia required initial intubation.
Lee et al. [18]	United Kingdom	Case report	1 patient with measles pneumonitis	Oral ribavirin 400 mg × 2 for 4 days	Patient recovered and discharged from hospital.	Immunocompetent adult.
Mutoh et al. [44]	Japan	Case report	Immunocompetent adult with severe measles pneumonia, and encephalitis complicated by ADEM	Corticosteroids, IVIG, vitamin A, plasma exchange	Clinical recovery.	Corticosteroid-based therapy may be beneficial for severe measles encephalitis in adults.
Caraffa et al. [45]	Italy	Retrospective multicenter observational study	113 hospitalized measles patients (59 adults, 54 children ≤16 years)	Systemic corticosteroids in 39 adult patients	Corticosteroid-treated adults did not show worse clinical outcomes c/w not treated.	Systemic corticosteroids may be safely used in selected adult measles cases, although no clear therapeutic indication has been established.
Suter et al. [46]	Switzerland	Case report	Adult with severe measles pneumonitis presenting as obstructive bronchiolitis	Day 1: 80 mg iv methylprednisolone Day 2–7: 50 mg prednisolone po, continuous tapering thereafter	Full clinical recovery with normalization of lung function.	Corticosteroids may be effective in treating severe measles pneumonitis in adults.

ref: reference; iv: intravenous; g: gram; LD: loading dose; MD: maintenance dose; IU: international unit; mg: milligram; ADEM: acute disseminated encephalomyelitis; IVIG: intravenous immunoglobulins; c/w: compared with; po: per os.

**Table 4:** Articles presenting data on the treatment of humans with SSPE, 2015–3 June 2025.

Study [Ref]	Country	Type of Study	No and Characteristics of Subjects	Therapeutic Agent	Findings	Comments
Miyazaki et al. [22]	Japan	Case series	3 patients with SSPE	Intraventricular ribavirin (1–4 mg/kg/day) and IFN-α (1.5–6 × 10^6^ IU/mL/day) for 10–14 days (intermitted administration at 10–21 days intervals)	Temporary relief of clinical symptoms.	Target ribavirin concentration in CSF: 50–200 μg/mL No severe side effects
Kwak et al. [32]	Korea	Case report	1 patient with SSPE	Intraventricular IFN-α therapy (1 × 10^6^ U) twice a week for 13 years, and inosiplex for 5 years)	Clinical improvement, neurological stabilization.	One episode of bacterial meningitis
Sonoda et al. [33]	Japan	Case–control	1 patient with SSPE	Intraventricular IFN	Prevention of deterioration.	14 years follow-up
Le Cras et al. [34]	France	Case report	1 patient with SSPE	Intrathecal IFN-α2b (3 × 10^6^ U/m^2^ per week for 1 month, then per month) for a total of 6 injections and inosiplex	Stabilized clinical condition.	None
Schmitz et al. [41]	Latvia	Case report	1 patient with SSPE	Three courses (each 14 days) of remdesivir	Τransient clinical improvement, fatal outcome.	None
Gebara et al. [42]	Israel	Case report	1 patient with SSPE	Remdesivir plus IVIG, intrathecal IFN-α	Remdesivir failed to halt measles-associated macular necrosis but may have prevented disease progression in the contralateral eye and CNS.	The patient had also macular necrotizing retinitis

SSPE: subacute sclerosing panencephalitis; ref: reference; mg: milligram; kg: kilogram; IFN: interferon; IU: international unit; mL: milliliter; CSF: cerebrospinal fluid; μg: microgram; m^2^: square meter; IVIG: intravenous immunoglobulins; CNS: central nervous system.

## Data Availability

Data derived from public domain resources.
